# Discrimination of Stereoisomers by Their Enantioselective Interactions with Chiral Cholesterol-Containing Membranes

**DOI:** 10.3390/molecules23010049

**Published:** 2017-12-25

**Authors:** Hironori Tsuchiya, Maki Mizogami

**Affiliations:** 1 Department of Dental Basic Education, Asahi University School of Dentistry, Mizuho, Gifu 501-0296, Japan; 2 Department of Anesthesiology and Reanimatology, University of Fukui Faculty of Medical Sciences, Eiheiji-cho, Fukui 910-1193, Japan; makikai@u-fukui.ac.jp

**Keywords:** stereoisomer, discrimination, enantioselective membrane interaction, chiral membrane, cholesterol

## Abstract

Discrimination between enantiomers is an important subject in medicinal and biological chemistry because they exhibit markedly different bioactivity and toxicity. Although stereoisomers should vary in the mechanistic interactions with chiral targets, their discrimination associated with the mode of action on membrane lipids is scarce. The aim of this study is to reveal whether enantiomers selectively act on chiral lipid membranes. Different classes of stereoisomers were subjected at 5–200 μM to reactions with biomimetic phospholipid membranes containing ~40 mol % cholesterol to endow the lipid bilayers with chirality and their membrane interactions were comparatively evaluated by measuring fluorescence polarization. All of the tested compounds interacted with cholesterol-containing membranes to modify their physicochemical property with different potencies between enantiomers, correlating to those of their experimental and clinical effects. The rank order of membrane interactivity was reversed by changing cholesterol to C3-epimeric α-cholesterol. The same selectivity was also obtained from membranes prepared with 5α-cholestan-3β-ol and 5β-cholestan-3α-ol diastereomers. The opposite configuration allows molecules to interact with chiral sterol-containing membranes enantioselectively, and the specific β configuration of cholesterol’s 3-hydroxyl group is responsible for such selectivity. The enantioselective membrane interaction has medicinal implications for the characterization of the stereostructures with higher bioactivity and lower toxicity.

## 1. Introduction

Since Louis Pasteur first separated two ammonium tartrate isomers in 1848, chiral recognition to discriminate stereoisomers has been one of major subjects in medicinal, pharmaceutical, and biological chemistry because enantiomers exhibit marked differences in bioactivity and toxicity. Biocompounds prefer only a single enantiomer, homochirality, as exemplified by natural amino acids and sugars composed of l-isomers and d-isomers, respectively. One enantiomer is preferable to its counterpart and a racemic mixture for greater efficacy and safety, leading to the advantage of using a pure single enantiomer [1].

**Figure 1:**
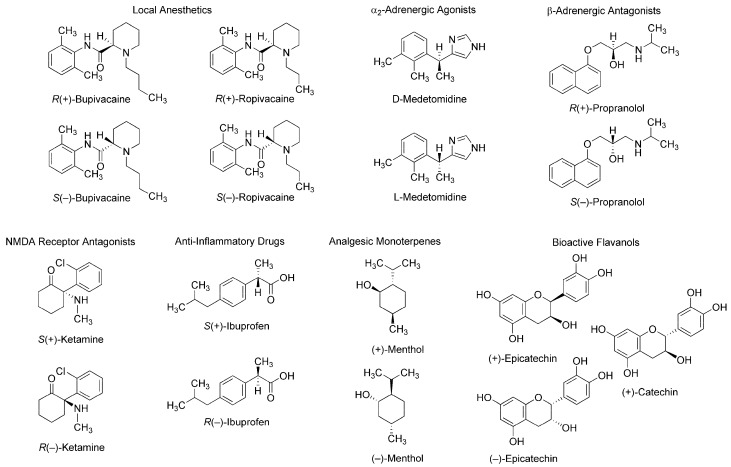
Stereoisomers with stereospecific bioactivity and toxicity. NMDA: *N*-methyl-d-aspartate.

Such stereospecific features are seen in different classes of compounds: local anesthetics [2], α_2_-adrenergic agonists [3], β-adrenergic antagonists [4], *N*-methyl-d-aspartate (NMDA) receptor antagonists [5], anti-inflammatory drugs [6], analgesic monoterpenes [7], and green tea flavans [8] (Figure 1). For example, *R*(+)-bupivacaine and *S*(−)-bupivacaine are distinguished in Na^+^ channel-blocking and local anesthetic activity [9] and *R*(+)-bupivacaine is more potent in cardiotoxicity than *S*(−)-bupivacaine and *rac*-bupivacaine [2]. Enantiomeric discrimination is critical in medicinal chemistry and the pharmaceutical industry to specify molecular structures with higher activity and lower toxicity. Chiral recognition is performed by various methodologies based on physicochemical and immunological techniques with chiral selectors such as crown ethers, polysaccharides, antibiotics, and antibodies [10,11]. Methodologies based on physicochemical techniques most commonly employ chromatography using chiral stationary phases or chiral derivatization, and methodologies based on immunological techniques, an enantioselective immunoassay. It is essentially desirable in medicinal chemistry to discriminate between enantiomers in association with their mode of action. However, such discrimination has been poorly studied.

**Figure 2:**
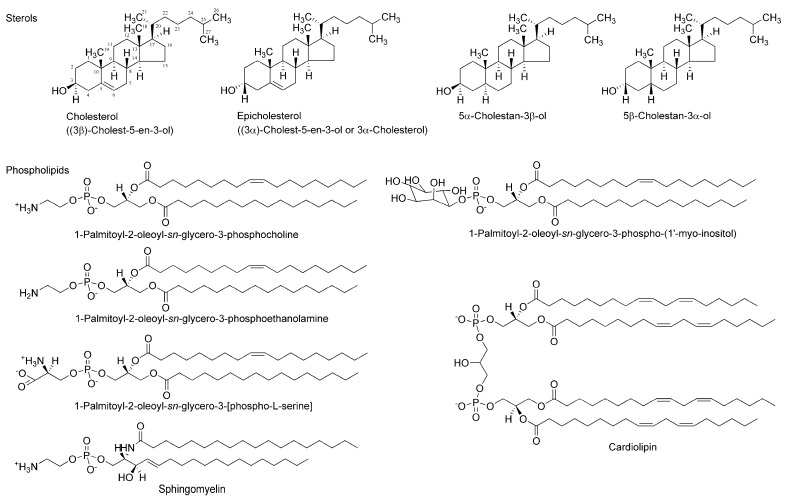
Membrane-constituting chiral phospholipids and sterols.

Two enantiomers absolutely differing in spatial configuration should behave differently in chiral environments, such as a biomolecular system. Since a number of drugs and compounds of biological origin target functional proteins, such as receptors, ion channels, and enzymes, that are entirely made up of only l-amino acids, their specific effects based on their stereochemical structure have been exclusively interpreted or theorized by discriminable spatial relationships of enantiomers in the asymmetric environments of receptor-, channel-, and enzyme-constituting chiral proteins. Membrane-constituting phospholipids and cholesterol naturally exist in only one enantiomer. Therefore, lipid bilayer membranes could also act as the enantioselective selectors or screens for stereoisomers [12]. A phospholipid’s glycerol backbone and cholesterol have one or more chiral centers (Figure 2), giving the possibility that enantioselective interactions may occur in a membranous lipid phase as well as in a proteinous phase [13]. Almost all of the molecules acting on ion channels, enzymes, and receptors possess amphiphilic structures that provide them with the property to interact with membrane lipids [14]. It is presumable that chiral lipids possibly interact preferentially with molecules with the same chirality or stereochemical configuration to change membrane physicochemical properties, such as fluidity [15,16]. If the induced changes are different between enantiomers, they would be a useful index to discriminate stereoisomers (enantiomers and racemates). Compared with the enantioselective binding to or affinity for functional proteins, membrane lipids have not been fully investigated about the stereospecific aspects of molecular interaction except for one report of Zunino et al. [17].

The aim of this study is to shed light on the property of chiral lipid membranes to discriminate stereoisomers. For this purpose, we comparatively determined the interactions of enantiomers with biomimetic membranes consisting of phospholipids and sterols. Here, we report that chiral sterols produce the enantioselective membrane interactions, for which the specific configuration of cholesterol’s 3-hydroxyl group is responsible. Our findings could help to specify and design molecular structures with higher bioactivity and lower toxicity.

## 2. Experimental

### 2.1. Materials

Stereoisomers of bupivacaine, ropivacaine, and medetomidine were supplied by Maruishi Pharmaceuticals (Osaka, Japan), AstraZeneca (Södertälje, Sweden), and Orion Corporation (Espoo, Finland), respectively. Single enantiomers and a racemic mixture of propranolol, ketamine, ibuprofen, and menthol were purchased from Sigma-Aldrich (St. Louis, MO, USA), and (−)-epicatechin, (+)-epicatechin, and (+)-catechin from Kurita (Tokyo, Japan). 1-Palmitoyl-2-oleoyl-*sn*-glycero-3-phosphocholine (POPC), 1-palmitoyl-2-oleoyl-*sn*-glycero-3-phosphoethanolamine (POPE), 1-palmitoyl-2-oleoyl-*sn*-glycero-3-[phospho-l-serine] (POPS), 1-palmitoyl-2-oleoyl-*sn*-glycero-3-phospho-(1’-myo-inositol) (POPI), sphingomyelin ((2S,3R,4E)-2-acylaminooctadec-4-ene-3-hydroxy-1-phosphocholine, SM), and cardiolipin (1,3-*bis*(*sn*-3’-phosphatidyl)-*sn*-glycerol, CL) were from Avanti Polar Lipids (Alabaster, AL, USA). Cholesterol ((3β)-cholest-5-en-3-ol or 3β-cholesterol) and epicholesterol ((3α)-cholest-5-en-3-ol or 3α-cholesterol) were obtained from Wako Pure Chemicals (Osaka, Japan) and Steraloids (Newport, RI, USA), respectively, and 5α-cholestan-3β-ol and 5β-cholestan-3α-ol from Sigma-Aldrich. 1,6-Diphenyl-1,3,5-hexatriene (DPH), 2-(9-anthroyloxy)stearic acid (2-AS), 6-(9-anthroyloxy)stearic acid (6-AS), 9-(9-anthroyloxy)stearic acid (9-AS), 12-(9-anthroyloxy)stearic acid (12-AS), and 16-(9-anthroyloxy)palmitic acid (16-AP) were from Molecular Probes (Eugene, OR, USA). Dimethyl sulfoxide (DMSO), ethanol of spectroscopic grade (Kishida; Osaka, Japan), and water of liquid chromatographic grade (Kishida) were used for preparing reagent solutions. All other chemicals were of the highest analytical grade available commercially.

### 2.2. Biomimetic Membranes

Biomimetic membranes labelled with DPH were prepared with Avanti’s enantiopure phospholipids of l-isomer form to be unilamellar vesicles suspended in buffer according to previous methods [18,19] with some modifications as follows. The appropriate amount of phospholipids and sterols dissolved in chloroform was placed in a glass pear-shaped flask and evaporated to remove the solvent completely with constant rotation of the flask so as to deposit a uniform film over the flask’s bottom. The lipid film was dissolved with an ethanol solution of DPH, and an aliquot (250 µL) of the resulting solution was injected four times into 199 mL of 10 mM 4-(2-hydroxyethyl)-1-piperazineethanesulfonic acid buffer of pH 7.4 containing 125 mM NaCl and 25 mM KCl under stirring above the phase transition temperatures of phospholipids to be a final concentration of 12.5 µM for total lipids and 62.5 nM for DPH. The membrane phospholipid compositions were adjusted to have the constant molar ratio of POPC:POPE:POPS:POPI:SM = 25:16:3:3:3 to mimic neuronal membranes and POPC:POPE:POPS:POPI:SM:CL = 25:16:3:3:3:10 to mimic cardiomyocyte membranes either in the absence or presence of sterols. In order to evaluate the effects of cholesterol, epicholesterol, 5α-cholestan-3β-ol, and 5β-cholestan-3α-ol, their compositions were varied from 0 to 40 mol %, but with the constant relative molar ratio of phospholipids.

### 2.3. Membrane Interactions

All stereoisomers were dissolved with DMSO, and the solutions were applied to the membrane preparations so that their final concentrations ranged from 5 μM to 200 μM. The concentration of DMSO was adjusted to be 0.5% (*v*/*v*) of the total volume so as not to affect the fluidity of intact membranes. Control experiments were conducted with the addition of an equivalent volume of solvent DMSO. After the reactions at 37 °C for 45 min, DPH fluorescence polarization was measured at 360 nm for excitation wavelength and 430 nm for emission wavelength by an RF-540 spectrofluorometer (Shimadzu; Kyoto, Japan) equipped with a polarizer and a cuvette thermo-controlled at 37 °C. Polarization values were calculated by the formula (*I*_VV_ − *GI*_VH_)/(*I*_VV_ + *GI*_VH_) according to the method of Ushijima et al. [20], in which *I* is the fluorescence intensity and the subscripts V and H refer to the vertical and horizontal orientation of the excitation and emission polarizer, respectively. The grating correction factor (*G* = *I*_HV_/*I*_HH_) is the ratio of the detection system sensitivity for vertically and horizontally polarized light, which was used to correct the polarizing effects of a monochromator. Compared with controls, a decrease and an increase of fluorescence polarization mean an increase of membrane fluidity (inversely proportional to membrane ordering) and a decrease of membrane fluidity (inversely proportional to membrane disordering), respectively [21]. When evaluating the effects of sterols, the polarization changes (%) relative to control polarization values were used to compare the interaction potencies between different biomimetic membranes because the polarization values of control membranes change with varying membrane lipid compositions.

### 2.4. Specific Membrane Regions

Biomimetic membranes were prepared with Avanti’s enantiopure phospholipids of l-isomer form to have the lipid composition of 25 mol % POPC, 16 mol % POPE, 3 mol % POPS, 3 mol % POPI, 3 mol % SM, and 10 mol % CL plus 40 mol % cholesterol to be a final concentration of 12.5 µM for total lipids as described above. *R*(+)-Bupivacaine and *S*(−)-bupivacaine were dissolved with DMSO, and the solutions were applied to the membrane preparations at 50 μM for each. The concentration of DMSO was adjusted to be 0.5% (*v*/*v*) of the total volume so as not to affect the fluidity of intact membranes. Control experiments were conducted with the addition of an equivalent volume of solvent DMSO. After the reactions at 37 °C for 45 min, the membranes were labelled with 2-AS, 6-AS, 9-AS, 12-AS, or 16-AP (the molar ratio of *n*-AS(P) to total membrane lipids, 1:210) according to the previous method [22]. Fluorescence polarization was measured at 367 nm for excitation wavelength and 443 nm for emission wavelength as described above. *n*-AS(P) (*n* = 2, 6, 9, 12 and 16) selectively locate at a graded series of levels in lipid bilayers to reflect the gradient of fluidity from the surface to the center of membranes depending on an increase of *n*. Because the deeper regions of lipid bilayer membranes are more fluid than the superficial region, *n*-AS(P) polarization values decrease with increasing *n*. Therefore, the *n*-AS(P) polarization changes (%) relative to control polarization values were compared to specify the membrane region responsible for enantioselective interaction.

### 2.5. Statistical Analysis

The data were statistically analyzed by one-way ANOVA followed by a post hoc Fisher’s protected least significant difference (PLSD) test using StatView 5.0 (SAS Institute; Cary, NC, USA). All results are expressed as mean ± SEM (*n* = 8 for each experiment) and values of ** *p* <0.01 were considered statistically significant.

## 3. Results and Discussion

### 3.1. Membrane Interactions in the Absence or Presence of Cholesterol

**Figure 3:**
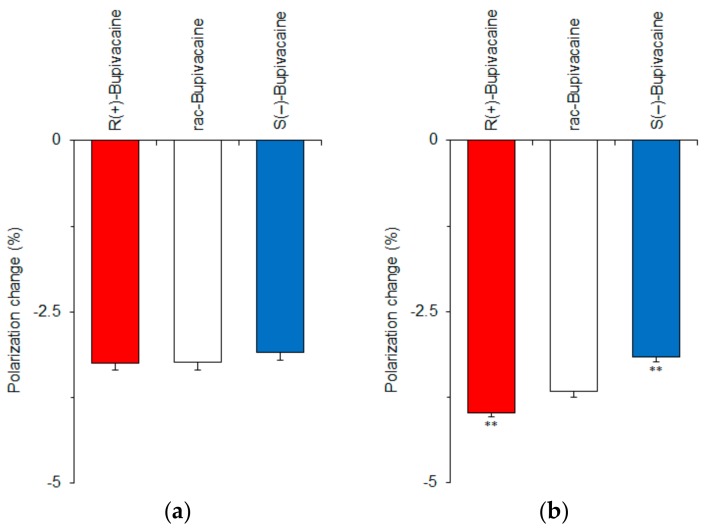
Interactions of bupivacaine stereoisomers (50 μM for each) with neuro-mimetic membranes in the absence (**a**) or presence (**b**) of 10 mol % cholesterol. ** *p* <0.01 compared with *rac*-bupivacaine.

We first verified whether membrane-constituting phospholipids or cholesterol produce the stereospecificity of membrane interactions by using *R*(+)-bupivacaine, *rac*-bupivacaine, and *S*(−)-bupivacaine. Bupivacaine-induced physicochemical changes of membranes were determined by measuring fluorescence polarization with DPH that has been most frequently used for membrane experiments [19,20,21]. This probe penetrates into lipid bilayers to align with phospholipid acyl chains, being subject to the rotational restriction imparted by membrane rigidity or order. All of the bupivacaine stereoisomers acted on neuro-mimetic membranes prepared with phospholipids in the absence or presence of cholesterol to increase the membrane fluidity as shown by decreases of DPH fluorescence polarization (Figure 3). However, bupivacaine enantiomers were not discriminated by the membranes consisting of phospholipids alone (Figure 3a). In previous studies, liposomes prepared with several phosphatidylcholines of l-isoform did not enantioselectively discriminate between *S*(+)-ibuprofen and *R*(−)-ibuprofen [23], whereas liposomes composed of 1,2-dipalmitoyl-*sn*-glycero-3-phosphocholine recognized l-amino acids differently from their d-enantiomers [24]. The reason why phospholipid membranes did not discriminate between enantiomers in our study may be that the chiral center is hidden inside the hydrocarbon tails of phospholipids [25].

Cholesterol has eight chiral centers: C-3, C-8, C-9, C-10, C-13, C-14, C-17, and C-20, which could contribute to the discrimination between enantiomers [13,26]. We prepared neuro-mimetic membranes with phospholipids plus 10 mol % cholesterol. Unlike phospholipid membranes, cholesterol-containing membranes enantioselectively interacted with bupivacaine stereoisomers with the potency being *R*(+)-bupivacaine > *rac*-bupivacaine > *S*(−)-bupivacaine (Figure 3b).

### 3.2. Effects of Sterols on Membrane Interactions

**Figure 4:**
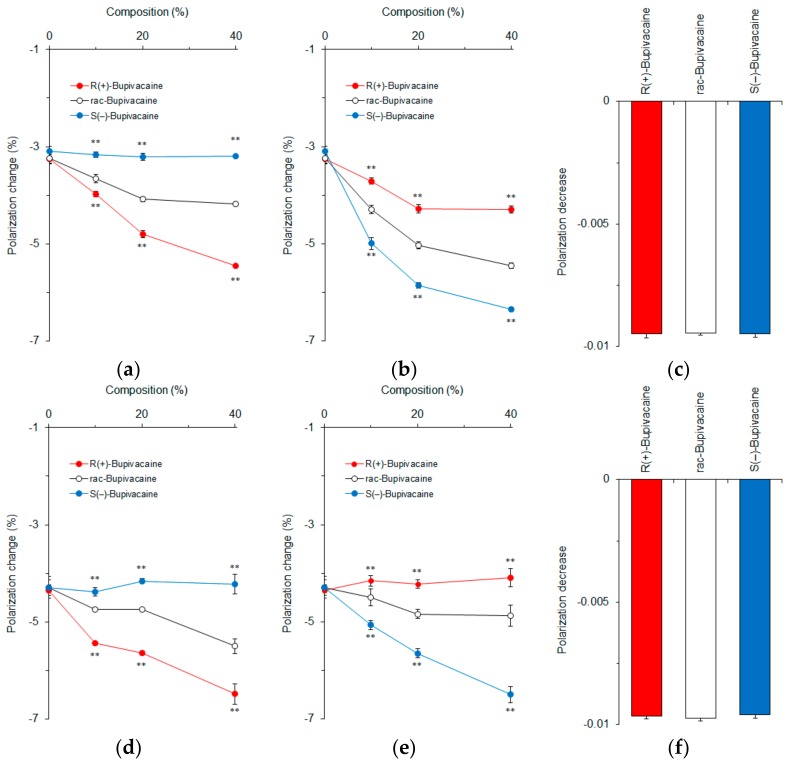
Effects of 0–40 mol % cholesterol (**a**); 0–40 mol % epicholesterol (**b**); 20 mol % cholesterol and 20 mol % epicholesterol (**c**); 0–40 mol % 5α-cholestan-3β-ol (**d**); 0–40 mol % 5β-cholestan-3α-ol (**e**); and 20 mol % 5α-cholestan-3β-ol and 20 mol % 5β-cholestan-3α-ol (**f**) on interactions of bupivacaine stereoisomers (50 μM for each) with cardiomyocyte-mimetic membranes. ** *p* <0.01 compared with *rac*-bupivacaine.

Epicholesterol is an unnatural epimeric form of cholesterol, differing from cholesterol only in the stereochemistry of the C-3 position. We prepared cardiomyocyte-mimetic membranes with either cholesterol or epicholesterol to investigate how these sterol epimers influence the membrane interactions of bupivacaine stereoisomers. Depending on an increase of cholesterol composition, bupivacaine enantioselectively interacted with the membranes with the potency being *R*(+)-bupivacaine > *rac*-bupivacaine > *S*(−)-bupivacaine as shown by different decreases of DPH polarization (Figure 4a). In contrast, ~40 mol % epicholesterol showed the rank order of membrane interactivity to be *S*(−)-bupivacaine > *rac*-bupivacaine > *R*(+)-bupivacaine (Figure 4b). The membranes prepared with equimolar cholesterol and epicholesterol (20 mol % for each) could not discriminate bupivacaine stereoisomers (Figure 4c). These results suggest that the configuration of a 3-hydroxyl group is very likely responsible for the enantioselective membrane interaction.

In order to confirm this, we used 5α-cholestan-3β-ol and 5β-cholestan-3α-ol to prepare cardiomyocyte-mimetic membranes. While cholesterol epimers are different from each other at only one chiral center (C-3), these cholestan diastereomers have opposite configurations at two equivalent chiral centers (C-3 and C-5). Bupivacaine interacted with membranes containing ~40 mol % 5α-cholestan-3β-ol with the potency being *R*(+)-bupivacaine > *rac*-bupivacaine > *S*(–)-bupivacaine in a cholestan composition-dependent manner (Figure 4d). In contrast, ~40 mol % 5β-cholestan-3α-ol showed the reversed rank order of membrane interactivity to be *S*(−)-bupivacaine > *rac*-bupivacaine > *R*(+)-bupivacaine (Figure 4e). The membranes prepared with equimolar 5α-cholestan-3β-ol and 5β-cholestan-3α-ol (20 mol % for each) had no ability to discriminate bupivacaine stereoisomers (Figure 4f).

Both membranes prepared with sterol epimers and diastereomers exhibited selectivity to either one of the bupivacaine enantiomers. The relative ratios of DPH polarization changes by *R*(+)-bupivacaine, *rac*-bupivacaine, and *S*(−)-bupivacaine were 1.77 ± 0.01, 1.35 ± 0.01, and 1.00 ± 0.01 in membranes containing 40 mol % cholesterol but 1.53 ± 0.05, 1.30 ± 0.03, and 1.00 ± 0.05 in membranes containing 40 mol % 5α-cholestan-3β-ol. Taken together, these comparative results indicate that the β configuration of a 3-hydroxyl group contributes to the enantioselective membrane interaction more significantly than different configurations of two chiral centers.

**Figure 5:**
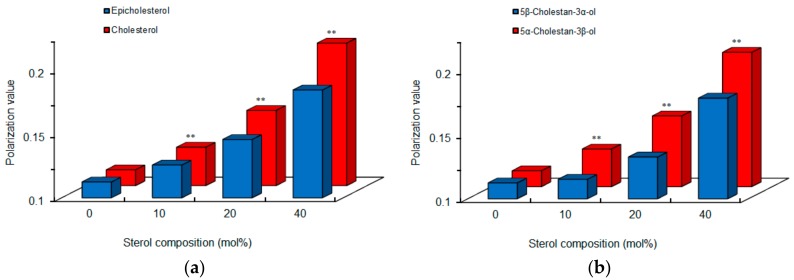
Comparisons of cardiomyocyte-mimetic membranes prepared with epimeric (**a**) and diastereomeric (**b**) sterols. ** *p* <0.01 compared with epicholesterol or 5β-cholestan-3α-ol.

Since chiral recognition is affected by the physicochemical properties of liposomal membranes [24], we compared intact cardiomyocyte-mimetic membranes consisting of 0–40 mol % epimeric or diastereomeric sterols. Cholesterol and 5α-cholestan-3β-ol showed larger DPH polarization values than epicholesterol and 5β-cholestan-3α-ol, respectively (Figure 5), indicating that a 3-hydroxyl group of the β configuration decreases membrane fluidity or increases membrane order more effectively than a 3-hydroxyl group of the α configuration. These results agree with previous reports that the effects of cholesterol to increase membrane order and condensation is greater than those of epicholesterol [27,28]. The membrane-ordering effects of sterols vary by α versus β configuration of a single hydroxyl group. The different polarization values also suggest a difference in membrane localization between epimeric sterols, that is, a 3α-hydroxyl group of epicholesterol protrudes into the water phase in lipid bilayers more than a 3β-hydroxyl group of cholesterol [28].

### 3.3. Membrane Interactions of Different Classes of Stereoisomers

**Figure 6:**
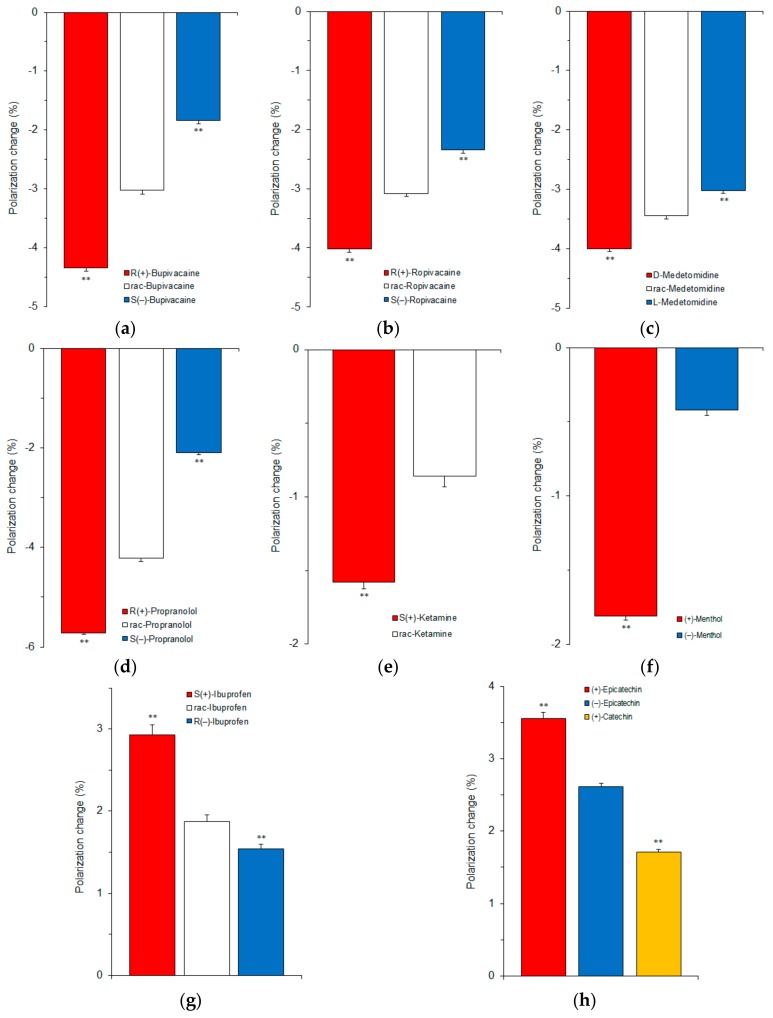
Membrane interactions of stereoisomers of 25 μM bupivacaine (**a**); 50 μM ropivacaine (**b**); 50 μM medetomidine (**c**); 50 μM propranolol (**d**); 50 μM ketamine (**e**); 50 μM menthol (**f**); 200 μM ibuprofen (**g**); and 100 μM catechin (**h**). ** *p* <0.01 compared with enantiomeric antipode, racemate, or epimer.

We revealed whether the enantioselective membrane interaction is applicable to stereoisomers other than bupivacaine. Different classes of stereoisomers were subjected at clinically or experimentally relevant concentrations of 25‒200 μM to the reactions with 40 mol % cholesterol-containing cardiomyocyte-mimetic membranes for local anesthetics and with 40 mol % cholesterol-containing neuro-mimetic membranes for others. All of the tested stereoisomers acted on biomimetic membranes to modify the membrane fluidity as shown by DPH polarization decreases (Figure 6a‒f) or increases (Figure 6g,h).

The relative potencies of membrane interaction were *R*(+)-bupivacaine > *rac*-bupivacaine > *S*(−)-bupivacaine, *R*(+)-ropivacaine > *rac*-ropivacaine > *S*(−)-ropivacaine, d-medetomidine > *rac*-medetomidine > l-medetomidine, *R*(+)-propranolol > *rac*-propranolol > *S*(−)-propranolol, *S*(+)-ketamine > *rac*-ketamine, (+)-menthol > (−)-menthol, *S*(+)-ibuprofen > *rac*-ibuprofen > *R*(−)-ibuprofen, and (+)-epicatechin > (−)-epicatechin. This is the first study to reveal that a variety of stereoisomers can be discriminated by interacting with chiral cholesterol-containing membranes. Zunino et al. [17] recently reported that (+)-neomenthol and (−)-neomenthol act on dipalmitoylphosphatidylcholine model membranes to induce different changes in DPH fluorescence anisotropy.

When comparing catechin stereoisomers (Figure 6h), the relative DPH polarization changes were 1.36 ± 0.04 and 1.00 ± 0.02 for (+)-epicatechin ((2*S*,3*S*)-*cis*-3,3’,4’,5,7-pentahydroxyflavan) and (−)-epicatechin ((2*R*,3*R*)-*cis*-3,3’,4’,5,7-pentahydroxyflavan), respectively, and 2.08 ± 0.06 and 1.00 ± 0.03 for (+)-epicatechin ((2*S*,3*S*)-*cis*-3,3’,4’,5,7-pentahydroxyflavan) and (+)-catechin ((2*R*,3*S*)-*trans*-3,3’,4’,5,7-pentahydroxyflavan), respectively. One configurational difference of C-2 epimers appears to be reflected in stereostructure-dependent membrane interactivity more strongly than the configurational differences of two chiral centers at the 2- and 3-position.

The relative ratios of DPH polarization changes by stereoisomers were 2.37 ± 0.03, 1.65 ± 0.04, and 1.00 ± 0.03 for *R*(+)-bupivacaine, *rac*-bupivacaine, and *S*(−)-bupivacaine (25 μM for each), respectively; 1.71 ± 0.02, 1.32 ± 0.02, and 1.00 ± 0.02 for *R*(+)-ropivacaine, *rac*-ropivacaine, and *S*(−)-ropivacaine (50 μM for each), respectively; 1.32 ± 0.02, 1.14 ± 0.02, and 1.00 ± 0.01 for d-medetomidine, *rac*-medetomidine, and l-medetomidine (50 μM for each), respectively; 2.73 ± 0.01, 2.01 ± 0.03, and 1.00 ± 0.02 for *R*(+)-propranolol, *rac*-propranolol, and *S*(−)-propranolol (50 μM for each), respectively; 1.84 ± 0.05 and 1.00 ± 0.09 for *S*(+)-ketamine and *rac*-ketamine (50 μM for each), respectively; 4.29 ± 0.06 and 1.00 ± 0.08 for (+)-menthol and (−)-menthol (50 μM for each), respectively; 1.90 ± 0.08, 1.22 ± 0.05, and 1.00 ± 0.02 for *S*(+)-ibuprofen, *rac*-ibuprofen, and *R*(−)-ibuprofen (200 μM for each), respectively; and 1.36 ± 0.03 and 1.00 ± 0.02 for (+)-epicatechin and (−)-epicatechin (100 μM for each), respectively.

It has been well-recognized that enantiomers are different in bioactivity and toxicity. However, there are few studies that determine quantitative differences of stereoisomers compared with qualitative ones. Bupivacaine and ropivacaine are used for regional nerve block, epidural anesthesia, and spinal anesthesia in surgery, but they adversely act on the cardiovascular system. In the guinea pig heart perfusion study of Graf et al. [29], the relative atrioventricular conduction time prolonged by *R*(+)-bupivacaine, *rac*-bupivacaine, and *S*(−)-bupivacaine was 1.54, 1.30, and 1.00 at 10 μM for each. Groban et al. [30] revealed that the comparative cardiotoxicity is 1.7 for *rac*-bupivacaine versus 1.0 for *S*(−)-bupivacaine in incremental escalating infusions of local anesthetics on open-chest dogs to the point of cardiovascular collapse. By comparing the electrocardiographic cardiotoxic effects in swine, Morrison et al. [31] demonstrated that the relative median lethal dose is 1.87 for *S*(−)-bupivacaine versus 1.00 for *rac*-bupivacaine. With respect to the effects on neuronal Na^+^ channels, the relative inhibition ratio of *S*(−)-bupivacaine to *R*(+)-bupivacaine is 1:3 in frogs [32] and 1:1.3–3 in rats [33]. Unlike d-medetomidine and *rac*-medetomidine, l-medetomidine has no significant sedative, analgesic, and cardiovascular effects [34]. Based on the sedation and analgesia scores of dogs undergoing propofol-isoflurane anesthesia, Kuusela et al. [35] concluded that intravenously administered d-medetomidine (0.2–20 mg/kg) is as safe and effective as *rac*-medetomidine (0.4–40 mg/kg). When lion tamarins were subjected to *rac*-medetomidine (20 μg/kg) and ketamine (10 mg/kg) anesthesia or to d-medetomidine (10 μg/kg) and ketamine (10 mg/kg) anesthesia, Selmi et al. [36] observed that the anesthetic quality and analgesia scores are greater in the d-medetomidine group. White et al. [37] showed that *S*(+)-ketamine is 3–5 times more potent in analgesia than *R*(−)-ketamine by a clinical trial. Evans et al. [38] indicated that the anti-inflammatory activity of *S*(+)-ibuprofen relative to *R*(−)-ibuprofen is 1.4 in experimental animals. Stoschitzky et al. [4] reported that *R*(+)-propranolol increases forearm blood flow in healthy subjects by brachial artery infusions, but not *S*(−)-propranolol. The β-adrenergic blocking activity resides in *S*(−)-propranolol, whereas the membrane stabilizing activity, in *R*(+)-propranolol [39]. Menthol enhances human γ-aminobutyric acid type A (GABA_A_) receptor currents and stimulates ligand binding to GABA_A_ receptors with the potency being (+)-menthol > (−)-menthol [7,40].

The rank orders of these bioactivities and toxicities agree with those of the membrane interactivity of all the tested stereoisomers. However, the quantitative comparisons of membrane-interacting potencies are not necessarily consistent with those of clinical and experimental effects, suggesting the possibility that modes of action other than the membrane interaction contribute to the enantioselectivity of stereoisomers.

### 3.4. Membrane Region Responsible for Enantioselective Interaction

**Figure 7:**
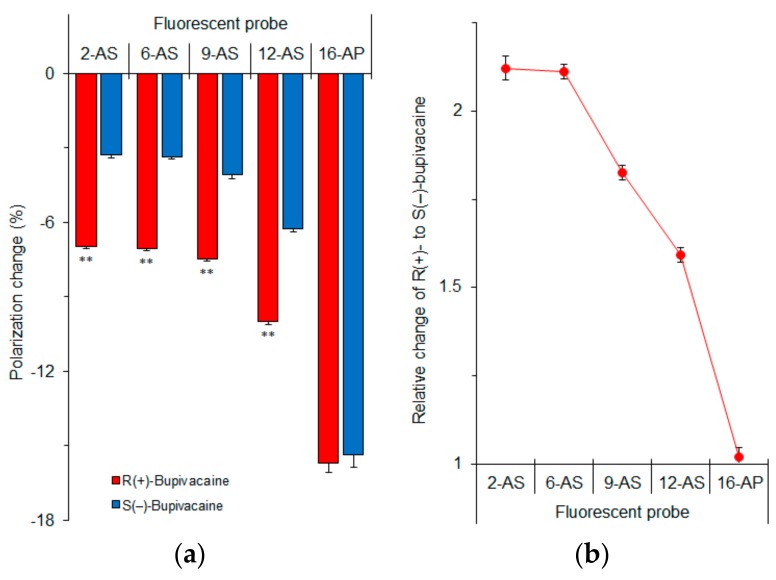
*n*-AS(P) polarization changes of cardiomyocyte-mimetic membranes induced by 50 μM bupivacaine enantiomers (**a**) and relative changes of *R*(+)-bupivacaine to *S*(−)-bupivacaine (**b**). ** *p* <0.01 compared with *S*(−)-bupivacaine.

In order to determine which membrane region is primarily responsible for the enantioselective interaction, we measured fluorescence polarization with *n*-AS(P) (*n* = 2, 6, 9, 12 and 16) after treating cardiomyocyte-mimetic membranes with bupivacaine enantiomers. These probes selectively locate at a graded series of levels in lipid bilayers from the surface to the center of membranes depending on an increase of *n*. Differences between *R*(+)-bupivacaine- and *S*(–)-bupivacaine-induced polarization changes increased with decreasing *n* of *n*-AS(P) (Figure 7a). The relative polarization changes of *R*(+)-enantiomers to *S*(–)-enantiomers were largest in 2-AS (Figure 7b). The enantioselective membrane interaction is most closely associated with the superficial region of lipid bilayers, not with the deeper region or membrane core. From these results, the discriminable membrane interaction between enantiomers is speculated to be as follows.

### 3.5. Possible Membrane Interaction

**Figure 8:**
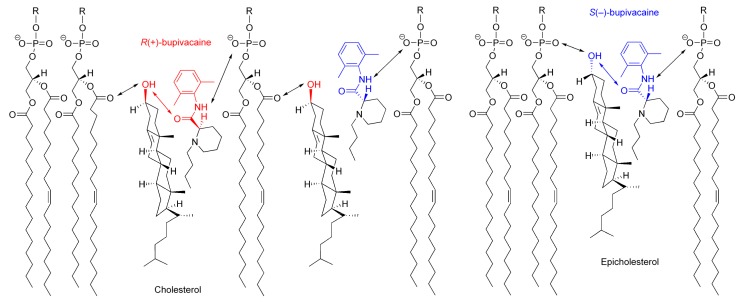
Possible interactions of bupivacaine enantiomers with cholesterol-containing membranes.

The possible interaction with chiral cholesterol-containing membranes is shown for bupivacaine enantiomers in Figure 8, comparing with epicholesterol-containing membranes.

Cholesterol orients in membranes with a polar hydroxyl group encountering the aqueous phase, a hydrophobic tetra-ring system buried in the hydrocarbon chains of phospholipids, and an isooctyl side-chain reaching the hydrophobic core of lipid bilayers [41,42]. However, cholesterol in lipid bilayers shows vertical localization different from epicholesterol. A 3β-hydroxyl group of cholesterol is positioned in the region of the phospholipid carbonyl groups, whereas a 3α-hydroxyl group of epicholesterol, in the region of the phospholipid phosphate groups [27]. Although the location and orientation of bupivacaine in membranes have not been detailed in the literature, other local anesthetics [43] suggest that an aromatic ring of bupivacaine could locate in the lipid/water interface of lipid bilayer membranes with its hydrophobic side chain oriented towards the hydrocarbon core. By referring to the membrane localization of articaine enantiomers [44], *R*(+)-bupivacaine is presumed to be located in the upper acyl chain/glycerol region of phospholipid bilayers, whereas *S*(−)-bupivacaine, close to the phospholipid polar head group. Since cholesterol protrudes less into the membrane/water interface than epicholesterol and *R*(+)-bupivacaine is intercalated deeper into phospholipid bilayers than *S*(−)-bupivacaine, cholesterol would interact more efficiently with *R*(+)-bupivacaine’s aminocarbonyl moiety that is located on stereochemically the same side as cholesterol’s 3β-hydroxyl group.

The opposite configuration allows enantiomers to be recognized differently through the interaction with another chiral molecule [45]. Chiral membrane lipids are considered to interact preferentially with molecules of the same chirality, producing higher selectivity to one enantiomer than its enantiomeric counterpart [15]. Among multiple (electrostatic and hydrophobic) interactions between molecules and membranes [23], the hydrogen bonding interaction plays a crucial role in chiral recognition [24]. Cholesterol can interact preferentially with *R*(+)-bupivacaine through the stereospecific hydrogen bonding of its 3β-hydroxyl group to the oxygen atom of bupivacaine’s carbonyl group in the *R* configuration, whereas epicholesterol, with *S*(−)-bupivacaine through the stereospecific hydrogen bonding of its 3α-hydroxyl group to the oxygen atom of bupivacaine’s carbonyl group in the *S* configuration.

Cholesterol and epicholesterol exhibit significant differences in biological properties, such as the inhibition of sarcoplasmic-endoplasmic reticulum calcium ATPase-2b activity [46], the reduction of large-conductance voltage/Ca^2+^-gated K^+^ channel activity [42], and the requirement for supporting serotonin_1A_ receptor activity [47]. Chiral cholesterol also physicochemically enables the cholesterol-bonded stationary phase to separate between enantiomers by liquid chromatography [48]. In the present study, cholesterol-containing membranes have discriminated stereoisomers by interacting with them enantioselectively, indicating that the β configuration of cholesterol’s 3-hydroxyl group is essential to chiral recognition as well as to biological functions. Espiritu et al. [49] recently reported the absolute requirement of such configuration for membrane-acting amphidinol 3. This antifungal agent exhibited high permeabilizing activity against and strong binding to cholesterol membranes, but neither activity against nor binding to epicholesterol membranes.

## 4. Conclusions

Stereoisomers can be discriminated by interacting with chiral cholesterol-containing membranes, and the rank orders of the membrane interactivity of enantiomers and racemates are consistent with those of their bioactivity and toxicity. The specific β configuration of cholesterol’s 3-hydroxyl group is responsible for such selectivity of membrane interaction. This is the first report to indicate that the opposite configuration allows molecules to interact with chiral membranes in an enantioselective manner and to apply such enantiomeric discrimination to different classes of stereoisomers. Our results have medicinal implications for the characterization of a single enantiomer with higher bioactivity and lower toxicity in association with one of the pharmacological mechanisms. Cholesterol is an essential membrane component not only to modulate the physicochemical property of membranes and the function of membrane-embedded proteins but also to enable the enantioselective interaction of stereoisomers with membranes.
